# Redefining CRP in tissue injury and repair: more than an acute pro-inflammatory mediator

**DOI:** 10.3389/fimmu.2025.1564607

**Published:** 2025-02-28

**Authors:** Marc Potempa, Peter C. Hart, Ibraheem M. Rajab, Lawrence A. Potempa

**Affiliations:** ^1^ Acphazin Inc., Deerfield, IL, United States; ^2^ College of Science, Health, and Pharmacy, Roosevelt University, Schaumburg, IL, United States

**Keywords:** pCRP, pCRP*, mCRP, hemostasis, inflammation resolution, tissue regeneration, therapeutic use

## Abstract

Most early studies investigating the role of C-reactive protein (CRP) in tissue damage determined it supported pro-hemostatic and pro-inflammatory activities. However, these findings were not universal, as other data suggested CRP inhibited these same processes. A potential explanation for these disparate observations finally emerged with the recognition that CRP undergoes context-dependent conformational changes *in vivo*, and each of its three isoforms – pentameric CRP (pCRP), modified pentameric CRP (pCRP*), and monomeric CRP (mCRP) – have different effects. In this review, we consider this new paradigm and re-evaluate the role of CRP and its isoforms in the tissue repair process. Indeed, a growing body of evidence points toward the involvement of CRP not just in hemostasis and inflammation, but also in the resolution of inflammation and in tissue regeneration. Additionally, we briefly discuss the shortcomings of the currently available diagnostic tests for CRP and highlight the need for change in how CRP is currently utilized in clinical practice.

## Introduction

The tissue repair process begins immediately after tissue damage and lasts for several weeks (1, 2). During this time, a series of biological processes occur that collectively staunch the injury (hemostasis) (3), stymie any invading pathogens (inflammation), (4), limit further damage (inflammation resolution and debris removal), (4, 5), and regenerate the tissue (angiogenesis, cellular proliferation, and tissue remodeling), (1, 4). While they overlap in practice, the various phases of the recovery process occur at roughly the following time frames: hemostasis, the first minutes to hours; inflammation, the first 72 hours; inflammation resolution, from 72 hours to ~1 week; and tissue regeneration and remodeling, ~1 week to ~1 month (1, 2).

For many years, C-Reactive Protein (CRP) was considered an important effector for only the earliest portions of the tissue repair response. This conclusion was driven by most biochemical and functional investigations of CRP determining that it potently supported pro-hemostatic and pro-inflammatory activities (6, 7). There was also a temporal logic to that argument, as plasma CRP concentrations increase up to 1000-fold during the pro-inflammatory phase and begin decreasing in tandem with the overall switch to inflammation resolution (8, 9). However, not all data were consistent with that interpretation. Some studies reported results in which CRP exhibited anti-inflammatory properties (10–15). Moreover, the 19-hour half-life of CRP means its levels are elevated above baseline even during the early tissue regeneration phase – a perplexing observation for something with strong pro-inflammatory potential (16). For a long time, these findings were difficult to reconcile and, to some extent, have limited the usefulness of CRP as a clinical tool and target.

Progress toward resolving these conflicting observations finally arrived with the recognition that CRP, in serum a very stable homo-pentameric macromolecule, undergoes conformational changes and dissociation at sites of inflammation *in vivo* (17). There had been *in vitro* observations to suggest a modified, monomeric version of CRP (mCRP) was the primary pro-inflammatory form of CRP (18–21), but evidence for the existence of mCRP *in vivo* had been difficult to obtain. The reasons for its delayed identification *in vivo* were multi-fold: for example, dissociation *in vitro* requires non-physiological amounts of heat, urea, or acidic environments (22–25); the exceptional insolubility of mCRP means it is only membrane-associated and/or -embedded *in vivo* (26–29); and, is inconsistently detectable on microvesicles in the serum of individuals without ongoing inflammatory disorders (27, 30–34). Nevertheless, improvements in techniques and reagents finally led to observations of pCRP dissociation *in vivo* in a rat model of myocardial infarction (17), its presence on circulating microvesicles in humans with inflammatory disorders (26, 27, 32–37), and its presence in human myocardial tissue and burn wounds (17, 38). A transitory intermediate form of CRP called pCRP* (pCRP star; also known as mCRPm) was identified shortly thereafter in which pCRP has undergone some conformational changes and exhibits some pro-inflammatory effector functions but has nevertheless not yet dissociated (39–42).

In this review, we distinguish between the three CRP isoforms and re-evaluate each of their potential roles in the tissue repair process. Specific isoforms of CRP will be described where possible, though many studies took place at a time where the need to differentiate the contributions of each isoform was not known or the ability to differentiate the isoforms was not readily possible. For these studies, the concentrations of CRP (low [i.e., non-saturating concentrations], pCRP*/mCRP; high, pCRP) and the time frame (≥0.5-2 hours, pCRP*/mCRP) in which results were observed provide potential ways to differentiate whether the reported effects were due to pCRP or pCRP*/mCRP (11, 39, 43). Nevertheless, there are inherent limitations to the discussion. Lastly, we briefly discuss the need for how CRP is used clinically to evolve in the wake of this new understanding of CRP bioactivity.

## CRP isoforms and their bioavailability

### Structure and general functions

Pentameric CRP is a compact, non-glycosylated, homo-polymeric molecule with a central void and radial symmetry (44). Each of the five monomers contains 206 amino acids and a single intramolecular disulfide bond, whereas the intermolecular interactions holding the pentamer together are non-covalent (44, 45). All monomers are oriented in the same direction, allowing pCRP to be conceptualized as two-sided (46). On one side is the binding face (or B-face), whose primary role is to bind phosphocholine (PC) (46–48). Though ubiquitously present, PC is normally buried within membranes and inaccessible to CRP. However, changes in membrane architecture due to lipid modification by enzymes (e.g., phospholipase A2) or reactive oxygen species (ROS) causes PC to ‘pop up’ and expose itself for CRP recognition (49–51). Upon exposure, it becomes a damage-associated molecular pattern (DAMP), an endogenous molecule containing a conserved motif the immune system utilizes to recognize abnormal situations and initiate an inflammatory response (52). Phosphocholine may also be found on Gram-positive bacterial cell walls (53), making it both a DAMP and pathogen-associated molecular pattern (PAMP; i.e., a conserved motif present on non-self organisms) (52). Interactions between CRP and PC are calcium-dependent and rely on CRP residues Phe-66 and Glu-81 (46). Notably, other DAMPs (e.g., oxidized low-density lipoprotein, histones, and fibronectin) and PAMPs (e.g., phosphoethanolamine [found on Gram-negative bacteria]) have also been identified as ligands for the CRP binding face (54–58).

On the reverse side of pCRP is the effector face, (also called the activating face or A-face), (46, 59). The most well-recognized binding partners for this half are the globular head of complement protein C1q and various Fc receptors (e.g., FcγRI [CD64], FcγRIIa [CD32a], FcγRIII [CD16], FcαRI [CD89]), (60, 61). Several other receptor binding partners have been suggested, including toll-like receptor 4 (TLR4), GPIbα, GPIIb/IIIa, CD31, CD36, integrin αvβ3, lectin-like oxidized low-density lipoprotein receptor-1 (LOX-1), and receptor activator of NF-κB ligand (62–70). While the binding site for C1q and the Fc receptors all overlap, the individual amino acids in CRP that facilitate binding to each ligand are distinct, even among the Fcγ receptors (FcγRs) (71). Importantly, three-dimensional models of the interaction between CRP and C1q suggest part of the interaction domain is inaccessible in the pentameric conformation (amino acids 199-206) (59). This implies pCRP is not inherently pro-inflammatory, instead requiring a conformational change into an alternative isoform for those activities to manifest. This is supported by the results of a clinical trial in which pCRP injected into healthy individuals did not elicit an inflammatory response (72). By extension, these results suggest environmental cues associated with ongoing inflammation are necessary to trigger conformational changes in pCRP, and the pro-inflammatory versions of CRP are amplifiers of inflammation rather than instigators. Unmodified pCRP may even have regulatory or anti-inflammatory activities, given *in vitro* observations of inhibitory effects on platelet, neutrophil, macrophage, dendritic cell (DC), and fibroblast activities in a dose-dependent manner (10–14, 73–78).

The pCRP* isoform is presumed to be an intermediate step between pCRP and its dissociation into mCRP (6, 39). Structurally, the pentameric assembly remains, but it has ‘relaxed’ sufficiently that the pro-inflammatory neoepitope (the aforementioned residues 199-206) is fully exposed (40, 41). At present, circumstances *in vivo* in which pCRP converts to pCRP* include ligand binding at regions of high membrane curvature and mildly acidic microenvironments such as those present at sites of inflammation (40, 79–82). Curved surfaces make PC more available, make hydrophobic regions of membrane lipids accessible, and expose binding sites on membrane-anchored proteins (81–83). Ultimately, the intermolecular interactions that hold pCRP subunits together undergo rearrangement resulting in exposure of the neoepitope (41). Alternatively (or additionally), acidic conditions can cause the protonation of histidine residues nearby the disulfide bonds within each CRP molecule (84). This alters the intramolecular hydrogen bonding network, causing structural changes in pCRP that again result in the exposure of the neoepitope.

Functionally, pCRP* stimulates the immune response by activating the classical complement pathway (39, 41). Interactions between CRP and C1q are primarily electrostatic in nature and demonstrate high avidity, making pCRP* the most potent CRP isoform at activating complement (41, 85). Of note, CRP-induced activation of the complement cascade biases it toward opsonization/phagocytosis as opposed to activation of the membrane attack complex (MAC)/cellular lysis (86, 87), thereby preventing excessive inflammation (87). Investigation of pCRP* activities beyond complement activation are limited due to its recent identification and the current limitations in experimentally distinguishing it from other isoforms. However, microvesicle-associated pCRP* can increase adhesion molecule expression on endothelial cells (41), and the overlap of the complement and FcγR binding sites implies pCRP* likely also stimulates FcγRs (71).

The terminal form of CRP is its monomeric form, mCRP. Dissolution of the pentamer occurs after newly exposed hydrophobic residues in pCRP* form interactions with the hydrophobic tails of lipids in membranes or with insoluble extracellular plaques in tissues (17, 22, 88). Thus, mCRP is found *in vivo* embedded within cellular membranes, associated with circulating microvesicles, or sequestered with insoluble components of the extracellular matrix (ECM) (17, 26–28, 38, 88, 89). The amino acids key to membrane-entry (residues 35-47, VCLHFYTELSSTR) preferentially interact with cholesterol, biasing mCRP membrane localization to lipid raft domains (28). Exposure of the cholesterol binding site is supported by reactive oxygen species (ROS) generated at sites of inflammation, presumably because the oxidative modifications to pCRP/pCRP* loosen its pentameric structure (79, 90). However, optimal exposure requires reduction of the intrachain disulfide bond, something it is primed to do in acidic conditions (79, 84, 89–91).

Pro-inflammatory activities have been described for mCRP in numerous settings and are discussed in detail in several recent reviews (6, 7, 92–99). In brief, mCRP promotes cellular chemotaxis and adhesion (14, 17, 18, 21, 68, 89, 100–105), augments platelet activation and aggregation (65, 70, 90, 106–108), and stimulates cytokine, ROS, and nitric oxide (NO) production (28, 38, 73, 79, 89, 102, 109–112). These effects are partially mediated through interactions with FcγRs, but not completely, as blockade of FcγRs does not completely abrogate the effects of mCRP (19–21, 113). Notably, mCRP potency is greater when the intramolecular disulfide bond in mCRP has been reduced (110). Monomeric CRP also retains the ability to interact with C1q and additionally interacts with negative regulators of complement activity (Factor H and C4-binding protein) (61, 114).

**Figure 1:**
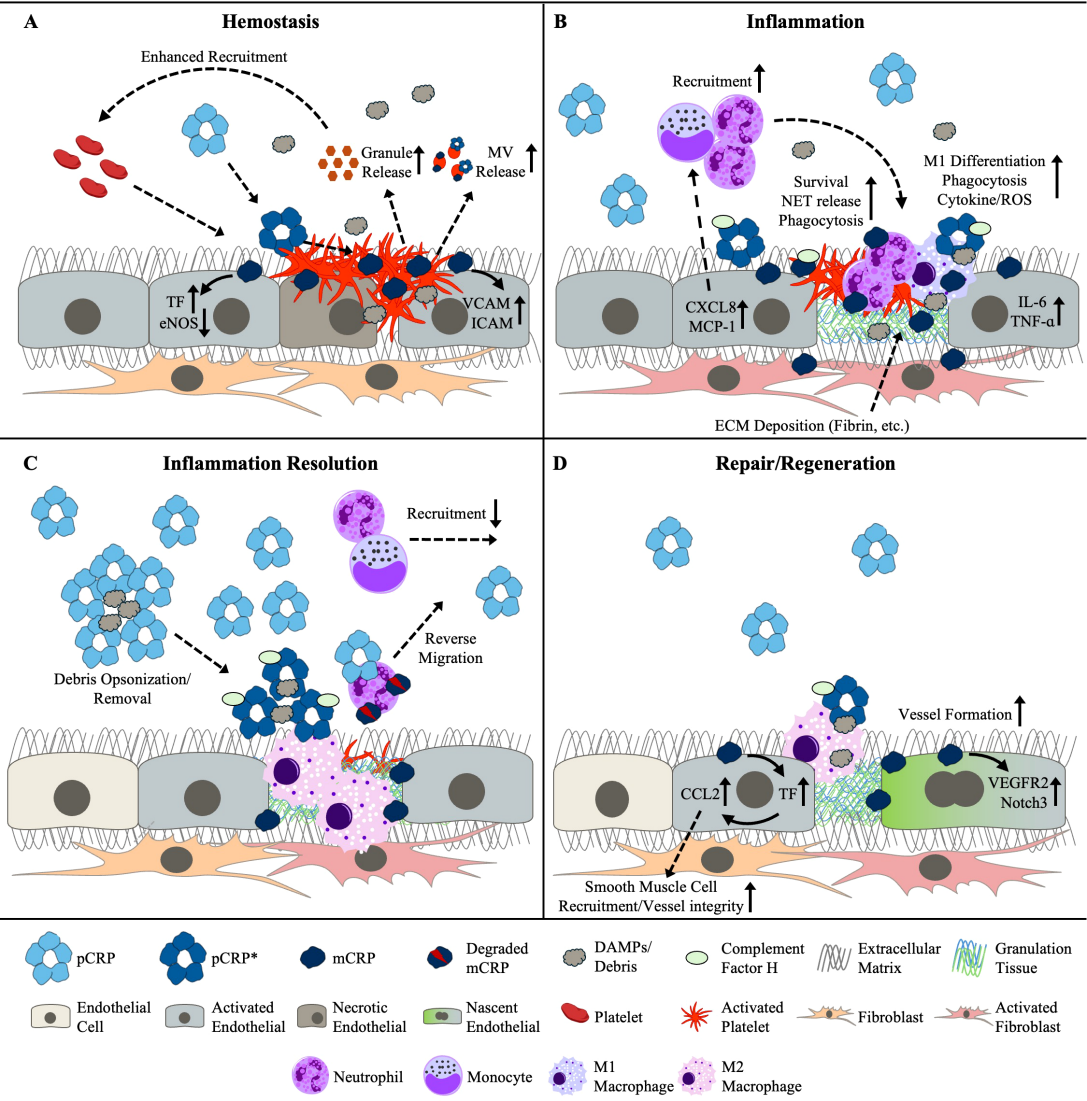
Reported and putative roles for the CRP isoforms on activities associated with **(A)** hemostasis, **(B)** inflammation, **(C)** the resolution of inflammation, and **(D)** tissue repair and regeneration. CRP, C-reactive protein; DAMPs, damage-associated molecular patterns ECM, extracellular matrix; eNOS, endothelial nitric oxide synthase; ICAM, intercellular adhesion molecule; IL-6, interleukin-6; MCP-1, monocyte chemoattractant protein-1; mCRP, monomeric CRP; MV, microvesicle; NET, neutrophil extracellular trap; pCRP, pentameric CRP; pCRP*, pCRP star; ROS, reactive oxygen species; TF, tissue factor; TNF-α; tumor necrosis-factor-alpha; VCAM, vascular cell adhesion molecule; VEGFR2, vascular endothelial growth factor receptor 2.

In summary, the long-appreciated role of CRP as an immune stimulant is now known to be attributable to pCRP* and mCRP, whereas pCRP is non- or anti-inflammatory. However, the bioactivities of CRP are not limited to impacting the inflammatory response. As we will shortly discuss, evidence has been accumulating to suggest CRP augments each additional phase of the tissue repair response: hemostasis, immune resolution, and tissue regeneration (**Figure 1**; **Table 1**).

**Table 1:** Reported pro-inflammatory and pro-resolution activities of CRP by cell type.

Cell Type	Reported effects of CRP
Platelets	
*Pro-inflammatory*	• Promotes adhesion and aggregation (65, 70, 90, 112, 138) • Enhances signaling through major platelet adhesion receptor GPIIb/IIIa and boosts responsiveness to other stimuli (65, 106) • Stimulates release of Factor V, vWF, Fibronectin, and high mobility group box 1 (65, 107, 108)
*Pro-resolution*	• Stimulates release of VEGF and PDGF (65, 108) • Inhibits aggregation (78)
Endothelial cells	
*Pro-inflammatory*	• Upregulates VCAM-1 and ICAM-1 expression (21, 28, 101, 105, 108, 141, 142, 152) • Promotes IL-6, CXCL8, and MCP-1 production and release (21, 28, 156, 159) • Increases surface expression of Tissue Factor (43, 145–148) • Inhibits endothelial nitric oxide synthase (143, 144) • Disrupts endothelial barrier integrity (105, 182, 193)
*Pro-resolution*	• Induces proliferation and tube formation (124, 145, 183, 194) • Upregulates VEGF receptor 2 and Notch3 expression (183, 187) • Regulates VE-cadherin and N-cadherin expression (187) • Upregulates thrombomodulin and downregulates vWF (142)
Smooth muscle cells	
*Pro-inflammatory*	• Increases surface expression of Tissue Factor (145, 149) • Stimulates expression of IL-6, MCP-1, and TNF-α (63, 167, 195) • Upregulates matrix metalloproteinase expression (196, 197)
*Pro-resolution*	• Promotes migration and proliferation (145, 198)
Neutrophils	
*Pro-inflammatory*	• Upregulates CD11b/CD18 expression and promotes infiltration (18, 104) • Increases nitric oxide and reactive oxygen species production (20, 102) • Enhances phagocytosis of debris (11, 113) • Promotes NET formation (107, 152, 162) • Delays apoptosis (19)
*Pro-resolution*	• Inhibits neutrophil chemotaxis and adhesion (11, 12)
Monocytes/Macrophages	
*Pro-inflammatory*	• Promotes differentiation into M1 macrophages and foam cells (111, 125, 169) • Augments expression of TNF-α, IL-1β, IL-6, and CXCL8 (15, 112, 152, 168) • Upregulates CD11b/CD18 expression and promotes recruitment (14, 68, 89, 101, 112, 147) • Increases nitric oxide and reactive oxygen species production (38, 73, 109, 152) • Enhances clearance of necrotic and apoptotic cells (38)
*Pro-resolution*	• Upregulates expression of LXRα (15) • Induces VEGF and IL-10 expression (176, 188) • Suppresses nitric oxide production (73) • Prevents conversion to foam cells and facilitates M2 polarization (77, 170)
Conventional dendritic cells	
*Pro-inflammatory*	• Promotes maturation of immature dendritic cells (199, 200) • Increases production of TNF-α and IL-12 (199)
*Pro-resolution*	• Suppresses stimulation of T cells (75, 201, 202) • Inhibits IFNα production in response to TLR ligands (13) • Drives formation of myeloid-derived suppressor cells (74)
Plasmacytoid dendritic cells	
*Pro-resolution*	• Suppresses IFNα production to autoantigens (203)
Mast cells	
*Pro-inflammatory*	• Promotes histamine release (204)
Fibroblasts	
*Pro-inflammatory*	• Increases IL-6, CXCL8, and VCAM-1 production (205, 206)
*Pro-resolution*	• Inhibits migration (76)

CRP, C-reactive protein; CXCL8, C-X-C motif ligand 8; HMGB1, high mobility group box 1; ICAM-1, intercellular adhesion molecule-1; IFNα, interferon alpha; LXRα, liver X receptor alpha; MCP-1, monocyte chemoattractant protein-1; NET, neutrophil extracellular trap; PDGF, platelet-derived growth factor; TLR, toll-like receptor; TNF-α, tumor necrosis factor alpha; VCAM-1, vascular cell adhesion molecule-1; VEGF, vascular endothelial growth factor; vWF, von Willebrand Factor.

### CRP bioavailability

In the absence of ongoing inflammation, the steady-state concentration of pCRP in blood is <1 to 3 mg/L (115–117). Circulating pCRP is produced by hepatocytes, though extrahepatic macrophages, lymphocytes, endothelial cells, adipocytes, and smooth muscle cells can express CRP (98, 118). It is unknown if non-hepatic CRP is secreted as pCRP or acts as an autocrine factor. Information on the steady-state levels of pCRP* and mCRP in the blood is limited. The pro-inflammatory CRP isoforms have been detected on microvesicles, but most efforts to quantify pCRP*/mCRP concentrations in the serum of individuals without inflammatory disorders place its concentration from undetectable (<1) to 25 ng/mL (26, 27, 33–36, 119–123). Outside of the blood, immunohistochemical staining finds pCRP*/mCRP to be present in arterial plaques and areas surrounding recently damaged vascular tissue (89, 99, 103, 124–126).

During the early stages of an inflammatory response, hepatocytes respond to elevated levels of interleukin (IL-6) and IL-1β by releasing pre-existing stores of pCRP and dramatically increasing production of new pCRP (127–129). Serum concentrations of pCRP can rise to over 500 mg/L within the first 72 hours of the response (9, 130). Similarly, microvesicle-associated CRP levels significantly increase during acute inflammatory events (34, 120, 123). Both pCRP and mCRP levels remain elevated in chronic conditions (27, 31–33, 35–37, 119, 121), with multiple reports finding a direct correlation between mCRP levels and disease severity (31, 32, 35–37, 131). This was in contrast to pCRP concentrations, which were not consistently predictive. Relatedly, there is disagreement about whether a correlation exists between mCRP and pCRP concentrations. Among 12 studies reporting correlations included in this review, nine found a lack of significant correlation (27, 30–34, 36, 37, 120–122, 131, 132).

Once secreted, the half-life of pCRP is ~19 hours (16, 117). Its rate of disappearance is independent of its plasma concentration (16), making the measured pCRP concentrations a reflection of recent synthesis rates and not changes in consumption/excretion. Due to the large amount produced and its relatively slow half-life, it is common to see elevated concentrations of circulating pCRP for more than a week after an inciting inflammatory event (133). The rate at which pCRP converts to pCRP* *in vivo* and the length of time before pCRP* dissociates into mCRP are unknown. *In vitro* observations found the neoepitope could be detected 30 minutes after treating cells with pCRP and that evidence of pentamer dissociation appeared approximately 90 minutes later (39). This timeframe roughly agrees with a second study that reported the appearance of mCRP at approximately 2 hours post application of pCRP (43). Information on the half-life of mCRP in humans is unavailable, both in the circulation and in tissues. However, data from mouse models revealed mCRP could be detected in tissues for three times longer than pCRP in the blood (134).

## CRP in tissue damage and repair

### Hemostasis

When bleeding occurs, multiple intertwined processes are initiated to close the wounded blood vessel (3, 135, 136). One process begins when platelets adhere to collagen in the exposed ECM. Binding activates the platelets, which recruit additional platelets that together coalesce into a primary plug. Secretions from activated platelets also provide a means for the activation a second clotting process, the intrinsic coagulation cascade. Platelet-derived polyphosphates provide a binding surface for coagulation Factor XII. Binding activates Factor XII and, after several additional steps, culminates in the activation of Factor X. In a third process, the extrinsic coagulation pathway, circulating coagulation Factor VII complexes with Tissue Factor (TF) expressed on the surface of smooth muscle cells and fibroblasts and this complex also activates Factor X. Activated Factor X combines with and activates Factor V, forming prothrombinase; prothrombinase converts prothrombin into thrombin; thrombin converts fibrinogen into fibrin; and Factor XIII, also activated by thrombin, covalently crosslinks fibrin molecules together. This fibrin mesh combines with the platelet plug to form a stable patch over the wound and prevent further blood loss (3, 135).

Foremost among the ways CRP boosts hemostatic processes is by enhancing platelet activation and aggregation (65, 70, 90, 106–108, 112, 137, 138). Platelets provide optimal conditions for the conversion of pCRP into pCRP* and mCRP. More specifically, the membranes of activated platelets contain an abundance of oxidized phospholipids and undergo membrane ‘ruffling,’ thus providing both exposed PC and regions of increased membrane curvature (89, 136). Indeed, half of all platelet-derived microvesicles have neoepitope-expressing CRP associated with them in people with an acute inflammatory condition (34), suggesting a close relationship between the two effectors *in vivo*. Functionally, mCRP enhances signaling through the major platelet adhesion receptor GPIIb/IIIa and boosts the responsiveness of platelets to other stimuli, such as adenosine diphosphate, epinephrine, and thrombin (65, 70, 90, 106). Platelets stimulated with mCRP release more of their granules (65, 108), which contain a variety of pro-coagulation factors (e.g., Factor V, von Willebrand Factor, fibronectin) and pro-repair factors (e.g., platelet-derived growth factor [PDGF], insulin-like growth factor-1, transforming growth factor [TGF]-β) (136). Increased secretion of High Mobility Group Box 1 (HMGB1) by platelets stimulated with mCRP has also been reported, which has downstream effects on neutrophils (107). The platelet scavenger receptor CD36 and adhesion receptor GPIIb/IIIa have been implicated in mediating some of the effects mCRP exerts on platelets (65, 70). Additionally, we note that platelets express ample amounts of FcγRIIa and TLR4, and there is substantial overlap between the effects observed with mCRP and those with FcγRIIa and TLR4 stimulation (139, 140).

Platelet activities are also affected by interactions between CRP and endothelial cells. Like with platelets, CRP can bind and dissociate on the membranes of endothelial cells at sites of inflammation (21). Stimulation of endothelial cells with mCRP leads to the upregulation of vascular cell adhesion molecule (VCAM)-1 and intercellular adhesion molecule (ICAM-1) (21, 28, 105, 108, 141, 142), the latter of which is a ligand for platelet GPIIb/IIIa (136). Thus, mCRP reinforces a major GPIIb/IIIa adhesion and signaling axis for platelets from both ends. CRP also supports platelet adhesion by inhibiting the expression and activity of endothelial nitric oxide synthase (eNOS) in endothelial cells (143, 144). Under normal conditions, endothelial cells produce nitric oxide to prevent unnecessary platelet aggregation and degranulation (3). By inhibiting eNOS, mCRP facilitates platelet adhesion and aggregation.

While its effects are less direct, CRP also impacts the extrinsic and intrinsic coagulation cascades. Endothelial cells and smooth muscle cells exposed to CRP upregulate TF (43, 145–149), thereby boosting the extrinsic coagulation pathway. Support for the intrinsic pathway stems from CRP-mediated activation of neutrophils. Neutrophils that swarm to the injury site generate structures called Neutrophil Extracellular Traps (NETs) (150). While the primary role of NETs is the capture of pathogenic organisms and cellular debris, they include polyanions that can also activate Factor XII (151). Among its numerous effects on neutrophils, mCRP has recently been suggested to promote NET formation, though this may be through an indirect mechanism in which neutrophils increase NETs in response to the HMGB1 secreted by platelets (107, 152, 162).

### Inflammation

The local immune response to tissue injury begins with the release of pro-inflammatory cytokines and DAMPs from damaged and dead cells (153–155). Nearby epithelial cells, endothelial cells, fibroblasts, mast cells, and tissue-resident macrophages respond to and amplify these signals to recruit nearby and circulating immune cells. For example, endothelial cells release IL-1β, IL-6, CXCL8 (i.e., IL-8), tumor necrosis factor (TNF)-α, and monocyte chemotactic protein (MCP)-1 to activate and attract immune cells and upregulate integrins like ICAM-1 and VCAM-1 to facilitate leukocyte adhesion to the site of damage (105, 108, 153–156). Aspects of the hemostasis response also contribute, with molecules such as thrombin stimulating cytokine secretion from local cells and platelets releasing pro-inflammatory chemokines and cytokines (136, 157).

A variety of innate immune cells, including neutrophils, monocytes, invariant NKT cells, mast cells, and plasmacytoid dendritic cells, respond to those pro-inflammatory cues and populate the wound site (154). Among them, neutrophils are the major effector for the first 24-48 hours post-injury, representing more than 50% of infiltrating leukocytes (4, 154). In general, their major activities are the secretion of antimicrobial substances (e.g., ROS) and the formation of NETs to capture and kill any potential pathogens. They also have a role as phagocytes, albeit only for smaller pieces of debris (150). After neutrophils, monocytes are the other major immune cell during the early response, peaking in number with a slight delay relative to neutrophils at approximately 72 hours post-injury (4). Responding monocytes initially differentiate into pro-inflammatory (i.e., M1) macrophages, release various pro-inflammatory cytokines and antimicrobial substances, and phagocytose pathogens, tissue debris, and apoptotic cells (4, 158). The specific contributions of the other cell types have been investigated more sparsely, though they are no less important to the timely repair of tissue damage (1).

The role of CRP in augmenting the acute inflammatory response is extensive and has been discussed at length by multiple other recent reviews (6, 7, 92–99). For brevity, we will briefly describe only a few key connections between CRP and neutrophils or monocytes/macrophages, and direct readers to the other reviews for more detailed information.

There is ample evidence linking CRP to enhanced neutrophil responses. First, CRP promotes neutrophil recruitment through its effects on endothelial cells and platelets. As described above, pCRP dissociates into mCRP on the surface of endothelial cells and promotes their activation. In doing so, mCRP boosts endothelial cell release of CXCL8 and upregulation of ICAM-1 (21, 28, 141, 159), a potent neutrophil chemoattractant and key ligand for neutrophil adhesion processes, respectively (4, 160). Similarly, CRP increases P-selection expression on platelets (65, 78, 138), which has a key role in neutrophil localization (21, 161). Neutrophils reciprocally upregulate CD11b/CD18 (Mac-1) after stimulation with mCRP (18, 162). In addition to its effects on neutrophil recruitment, stimulation of neutrophils with mCRP increases NO production, enhances phagocytosis of debris, delays their apoptosis, and has recently been demonstrated to be a potent inducer of NET formation (11, 19, 20, 107, 113, 152, 162). Some effects are downstream of interactions between mCRP and FcγRIIIb (19, 21), which is amply expressed on the neutrophil surface (163), whereas other effects may be downstream of FcαRI (164).

Monocyte and macrophage recruitment is similarly enhanced by CRP through the upregulation of MCP-1 expression in endothelial cells, and through the stimulation of receptors with which CRP is known to engage (21, 159, 165, 166), such as FcγRI, FcγRIIa, and toll-like receptor TLR4 (62, 63, 71, 167). Interactions between mCRP and monocyte (FcγRs) promote monocyte differentiation into M1 macrophages and contribute to the cellular metabolic reprogramming necessary for macrophages to perform their effector functions (17, 27, 111, 168–170). Cytokines released by monocytes/macrophages whose secretion has been augmented by mCRP include at least TNF-α, IL-1β, IL-6, and CXCL8 (63, 111, 112, 167, 169). Other effects of stimulating monocytes with mCRP include the upregulation of CD11b/CD18, increased NO and ROS production, and enhanced clearance of necrotic and apoptotic cells (14, 17, 73, 109, 111).

### Inflammation resolution

While inflammation is necessary for the elimination of pathogens and clearance of cellular debris, prolonged inflammation will stymie reparative activities (4). To prevent this, there are numerous ‘built-in’ mechanisms to ensure a timely resolution to inflammatory processes. For example, NETs catch cytokines and chemokines produced during the initial response, which results in their degradation by NET-associated proteases and a reduction in further effector cell recruitment (171). Activated neutrophils recruit monocytes and macrophages (172), and those macrophages subsequently contribute to suppressing neutrophil responses through efferocytosis and promoting neutrophil reverse migration (173, 174). Efferocytosis simultaneously promotes the conversion of M1 macrophages to the pro-resolution M2 phenotype that produce anti-inflammatory factors such as TGF-β and IL-10 (175). Overall, by approximately 72 hours post-injury, the inflammatory response to tissue injury should be ending and a pro-repair microenvironment forming.

There is evidence to suggest CRP has its own negative feedback mechanism. As noted, circulating pCRP concentrations may increase up to 1000-fold in the first 72 hours of an inflammatory response (9, 130). Interestingly, several *in vitro* observations have found high concentrations of pCRP to cause the opposite effects of pCRP*/mCRP or outright suppressive activities (10–13, 73–75, 78, 170). For instance, elevated pCRP concentration may help abate inflammation by suppressing the differentiation of pro-inflammatory DCs and driving the formation of myeloid-derived suppressor cells and M2-type macrophages (13, 74, 75, 170, 176). Moreover, whereas lower concentrations of CRP promote neutrophil chemotaxis and adhesion, higher amounts inhibit those activities (11, 12, 78, 177). At least for neutrophils, mCRP and pCRP may bind different receptors (178), ostensibly providing a mechanistic basis for these opposing effects. Notably, whereas pCRP is generally resistant to proteolysis, mCRP is susceptible to degradation by neutrophil-associated proteases and those peptides demonstrate dominant negative-like activities *in vitro* (177, 179, 180). Thus, mCRP binding sites may not be re-exposed after degradation of mCRP, which would also shift the balance of CRP activities toward those mediated by pCRP. Such mechanisms may contribute to the enigmatic process of neutrophil reverse migration (173).

Elevated CRP concentrations may also help limit inflammation by reducing and/or obscuring DAMPs. For example, CRP neutralizes extracellular histones from inducing endothelial cell cytotoxicity by outcompeting cell-associated binding partners that facilitate histone uptake (54). Furthermore, CRP prevents the activation of endothelial cells and macrophages by modified lipids if allowed to complex with those lipids prior to being added to cell cultures, suggesting a potential competitive inhibitory effect when in excess (14, 77). Thus, we propose that upregulation of CRP may serve as a mechanism by which an inflammatory response is curtailed through use of CRP as an “antigen sink.” The role of CRP as an opsonin of cellular debris is arguably also an anti-inflammatory mechanism of action, given the interaction of CRP with inhibitors of the MAC results in the clearance of inflammatory materials without inciting an inflammatory response (86). Higher concentrations ostensibly facilitate greater clearance, especially as the peak of CRP concentrations coincide with the peak of monocyte/macrophage infiltration.

Altogether, these findings suggest the up to 1000-fold increase in CRP concentration seen during the first 72 hours of a response may constitute an anti-inflammatory process rather than one meant to amplify inflammation. These anti-inflammatory effects may be achieved through at least two mechanisms: saturation of mCRP binding followed by the initiation of alternative anti-inflammatory interactions, and hiding/eliminating DAMPs/PAMPs by acting as an antigen sink. Further research into the anti-inflammatory properties of CRP are needed, especially as there is some evidence suggesting additional feedback mechanisms. For example, stimulation of macrophages through FcγRI by CRP upregulates expression of the inhibitory liver X receptor (LXR) alpha and specific ligands may lead to differing pro- or anti-inflammatory effects (15, 170, 176).

### Tissue regeneration and remodeling

The tissue regeneration and remodeling phase includes its own set of interdependent processes, which together account for the growth of new blood vessels (i.e., angiogenesis), the deposition of granulation tissue, the proliferation of parenchymal cells, and the remodeling of the tissue into a stable long-term structure (1, 4). Though conceptually these processes occur after hemostasis, inflammation, and inflammation resolution, considerable groundwork for them takes place during those earlier stages (4). For example, platelets secrete many pro-angiogenic factors (e.g., PDGF, vascular endothelial growth factor [VEGF], and TGF-β), stirring endothelial cells to proliferate and begin the formation of new capillaries (181). Proteases released by neutrophils free VEGF sequestered within the ECM and facilitate endothelial cell expansion into the wound (160). Histamine and trypase released by mast cells enhance fibroblast proliferation and the deposition of collagen (1, 4). Macrophages consume dead vasculature and secrete wound healing factors like arginase, TGF-β, VEGF, PDGF, and insulin-like growth factor (158). Indeed, there is an ever-growing list of interactions between the immune response to tissue damage and tissue regrowth.

Several observations now point toward mCRP being among the list of immune mediators to enhance tissue regeneration. Most of that evidence revolves around the effect of mCRP on neovascularization. For one, mCRP colocalizes with a marker of angiogenesis (i.e., endoglin [CD105]) in stroke patients (124, 182), suggesting a potential relationship *in vivo*. Results from *in vitro* wound healing assays support this, as aortic endothelial cells treated with mCRP exhibited greater vessel formation (124, 183). The upregulation of two critical receptors for vessel development, VEGF receptor 2 and Notch3, by endothelial cells after mCRP exposure offers a potential mechanism for this observation (183–185). Moreover, angiopoietins are upregulated downstream of Notch receptors and its production enhanced by hypoxia-inducible factor (HIF)-1α (184). CRP has also been shown to stimulate HIF-1α in a pro-angiogenic capacity (186), implying CRP both promotes Notch receptor expression and enhances signaling downstream of those receptors. There is also evidence to suggest mCRP contributes to both the formation and stability of neo-vessels by variably promoting or downregulating the expression of VE-cadherin and N-cadherin depending on co-stimulatory signals (187). The enhanced expression of TF by endothelial cells in the presence of mCRP adds another layer (43, 145–149), as TF activation increases endothelial cells secretion of CCL2, which recruits vascular smooth muscle cells that strengthen vessel integrity (43). Lastly, there are also indirect effects stemming from mCRP-mediated release of pro-angiogenic factors such as VEGF and PDGF (183, 186, 188).

The effect of CRP on other aspects of tissue regeneration and remodeling process has been investigated much more sparsely. There are likely effects on granulation tissue formation, since CRP has been reported to enhance the epithelial-to-mesenchymal transition (91). Conversely, other work has shown CRP can inhibit fibroblast migration (76), though this was again dose-dependent and so may represent another concentration-dependent negative feedback mechanism.

## In the clinic

At present, the only diagnostic tests for CRP measures plasma concentrations of the pCRP isoform (27). Clinicians have traditionally used results of those tests to report the presence of inflammation if the levels are above 10 mg/L (roughly 3- to 10-fold above baseline). However, such individual measurements cannot discern whether the inflammation is due to a chronic ongoing inflammatory event or a recent acute inflammatory event that has concluded. And because the amount of pCRP made varies from event to event and person to person (189, 190), single measurements are also unable to discern how long ago or how severe such an acute event might have been. Therefore, given the currently available diagnostic tests, we encourage physicians to measure pCRP levels multiple times with the understanding that its concentration should halve approximately every 19 hours (16, 117, 189, 191), excluding any potential effects of changes in treatment regimen. This minor change could at least assist clinicians in diagnosing the nature of a condition as acute or chronic.

Regardless, the more significant benefit to clinical practice would be the development of a routine clinical method for determining the abundance of the pro-inflammatory CRP isoforms (i.e., pCRP* and mCRP), as advances in the CRP field over the previous decade have confirmed these to be the potentially immunopathogenic forms of CRP (6, 96, 99, 192). Unfortunately, both the standard and high-sensitivity tests for pCRP are unable to detect pCRP*/mCRP and, critically, most findings have found there is no definitive correlation between serum concentrations of pCRP and pCRP*/mCRP (27, 30–34, 36, 37, 120–122, 132). This means there is no concrete means of discerning the amount of potentially immunopathogenic CRP from current standard practices. Of note, this potential absence of a relationship between the different isoforms may also explain the lack of agreement among studies investigating whether baseline pCRP levels predict the incidence of various cardiovascular conditions (6). There are putative correlations between CRP-positive microparticles (which ostensibly represents ligand-bound, neoepitope-exposed CRP) and C1q-positive microparticles, suggesting there may yet be surrogate methodologies in the absence of direct mechanisms; but even these relationships may be condition-specific (119, 120). Ultimately, it is imperative that routine, standardized assays are developed for the specific detection of pCRP*/mCRP. Only then can the relationship between CRP and underlying inflammatory diseases be clearly elucidated.

## Conclusion

The recognition that CRP undergoes context-dependent conformational changes *in vivo* has helped resolve long-standing contradictions in CRP research. Moreover, the distinct activities of pCRP, pCRP*, and mCRP have revealed the existence of a much more complex role for CRP in the biological response to tissue damage. Not only does CRP promote early hemostatic and inflammatory processes, but it also contributes to the resolution of inflammation and to angiogenesis. Moving forward, more efforts should be put toward defining the specific conditions in which each isoform is abundant, including considerations for factors such as the specific ligands available and cell receptor density. Toward that end, the development of standardized assays capable of detecting the pCRP* and mCRP isoforms is of paramount importance, as neither the general nor high-sensitivity CRP assays currently available have that ability. Such advances could also transform CRP from a general inflammatory marker into a more precise diagnostic tool, potentially enabling better monitoring of disease progression and therapeutic responses across a range of inflammatory conditions.
